# 

**Published:** 1860-03

**Authors:** 


### BUSINESS NOTICES.

VULCANITE, CORALITE, AMBER-BASE, ETC.

In the January No. of the Dental Register, notice was made of theVulcanite Patents; or rather the decision in favor of the American Hard-Rubber Company, against E. A. L. Roberts, in which I stated that I had taken some steps to investigate the matter, and might be able to say more in the February issue. I *never fail to redeem my promises*. In this case the promise was *contingent* upon whether I could obtain information of sufficient importance to place before the profession. I have not yet obtained such information, as it is a *slow* process to go behind the records of a court, in a distant city, for information.

In my recent travels, attending the meetings of Dental Associations in Indiana and Michigan, it was broadly hinted to me that letters had been issued from head quarters, to local and traveling agents of the American Hard-Rubber Co., bidding them be of good-cheer, that at the *earliest convenience* of those in power, quietus would be placed on "all infringers." Also, that in the next number of the New York Dental Journal, TOLAND would receive special attention, in an article giving him "*fitts!!*" If I have been misinformed I ask the gentleman's pardon. If my friend, Dr. Perin, or his able assistand, Frank Norton, do themselves justice in this matter, it will well repay any Dentist a year's subscription to the New York Dental Journal, for the privilege of perusing this promised article alone, and as my friend Perine would say (if he were present,) it would then become necessary to subscribe for the REGISTER, to see the reply.

Well, there are, I believe, only five dental periodicals published in the United States, and if every dentist would take all of them, he would not have any *too much information*, but enough of jesting—to the subject, which is naturally a serious one, because I might pursue a course myself which I would not advise my friends to follow, because if evil results should follow, they would naturally blame me, as no one likes to find fault with himself.

I am daily in receipt of letters (sometimes a dozen per day,) making inquiry as to the kind of aparatus used for vulcanite, coralite, and amber bases, and whether either can be used without a right or license. In reply to all inquiries, let me say, I will give you all the facts as nearly as I am possessed of them and let you judge for yourselves.

If any errors of fact, or any erroneous deductions are made, it will be my greatest pleasure to correct them on satisfactory information. Let the American Hard-Rubber Company speak out, and inform the profession precisely what their patent does and does not cover, how far material can be manufactured, bought and sold, or used and vulcanized, where their rights begin and where they end.

A patent is in itself presumptive evidense of its validity, until legally contested and *proved* to be *invalid*. The American Hard-Rubber Co., have two patents, re-issued May 18, 1858, which expire May 6, 1865. One for the hard product, or compound of india rubber and allied gums—the other for the process for producing the said product or substances. They have also obtained an injunction against Roberts for compounding and vending materials, and a verdict for damages. This strengthens the presumptive validity of their patent. It is therefore clear that only one of two courses can be pursued by those who wish to use the process and materials. The first to purchase a license, and with it the privilege to purchase their material at three dollars per pound. The other is, to go to work without a license, and purchase materials at higher prices, viz: five, eight, ten or fourteen dollars per pound, and take the risk of prosecution. Whether they will be prosecuted will depend on three things:

1st. Whether it becomes known to the Hard-Rubber Company, that the person is vulcanizing, and whether they feel satisfied that they can prove an infringement.

2d. Whether the person vulcanizing becomes an obstacle to the American Hard-Rubber Company, in the sale of licenses.

3d. Whether the person is sufficiently responsible to have costs and damages or either collected from him.

The maunfacturers of the various compounds, such as "Wheat's Compounds," "Improved Gum," "Enameled Gum," &c., claim that their compounds are *not* an infringment upon the Good Year Patents, and parties using them not liable to prosecution, and there is strong presumptive evidence to this effect, in the fact that the above named materials are manufactured in the city of New York, in close proximity to the head-quarters of the American Hard-Rubber Company's agency, and yet these parties are *unmolested* by any legal process.

If these are infringements, why are not they prosecuted, and enjoined from further manufacture and sale of materials.

THE AMBER BASE—comes next and claims *special* attention.

1st. By reason of the *color*, thereby being enabled so closely to represent the natural flesh color that gum teeth are not required, but *plain teeth* only are used.

2d. Being tasteless and free from odor, it is free from the objection frequently urged against the vulcanite base, viz : the disagreeable taste and odor arising from the combination of sulphur and india rubber.

3d. It is covered by four *distinct patents*, numbered and dated as follows : No. 19,916, April 13th, 1858 ; No. 24,544, June 28th, 1859 ; No. 24545, June 28th, 1859, and No. 25,957, November 1st, 1859. It will be seen that all except the first have been issued subsequent to the re-issue of the Good Year patent, and these cover every part of the process, viz : the compounding of materials, the use of *plaster moulds*, and process of curing or hardening or what is generally termed *vulcanizing*, *either by steam or any other kind of heat.* These patents must occupy precisely the same position as all others, viz : that the *patent is* in itself *presumptive evidence* of its *validity*. Why do the American Hard-Rubber Company not contest the validity of these patents. Dr. Diffenback has by circular and advertisements, *challenged* them to a *legal investigation.*

They probably reply that they do not claim *broadly* the manufacture of materials, but the constructing of plates or artificial denture therefrom. The fact is as before stated, they have two patents one for the *substance*, the other for the *process*, it is fair to infer that if they can not claim *bro dly* the *process*, they can not on the same principle claim *broadly* the *substance* obtained by the said process.

Those who purchase the amber compound, have a right to use it free from any tax or license. Those who wish the *exclusive* use for cities, counties and States, will have to purchase rights.

I am frequently asked, "is the amber base all right, has it strength, durability, &c.?" I can only reply that it is comparatively new, and my experience is limited, but from what I have seen of it, I believe there is no important difference in strength and durability between it and the vulcanite base, and am inclined to believe it will prove all that is claimed for it.

The American Hard-Rubber Company, sell their office-rights, for the residue of the time for which the patent was granted, for *one hundred dollars.* I will be pleased to receive the orders of those who wish an office-right, which shall be supplied to them on the most *liberal* terms.

With these facts before them, the profession can easily determine whether to purchase a license and use the American Hard-Rubber Company's Gum, or whether they will purchase only the apparatus, and use the other materials. The vulcanizer or apparatus is precisely the same, whether for hard-rubber, coralite or amber. The process is also in all respects similar, except the degree of *heat* and length of *time* required to produce the hardening, which is different for the different gums.

I am prepared to supply all kinds of materials, tools and machines.

Amber Base, $14 per pound, vulcanizes at 310° Fahrenheit. There is also a new article at $4 per pound.

| | | |
|---|---|---|
| Enameled Gum....................... | $10 | These vulcanize in six hours at a heat of 300° Fahrenheit, first hour, gradually increased so that the last hour is at 340° F. These may also be vulcanized in 3 or 4 hours at 326° or 330 ° F. |
| 1st Quality Pressed India Rubber ..... | 8 | |
| 1st " " Gutta Percha ...... | 8 | |
| Wheat's Compound.................. | 8 | |
| 2d Qnality India Rubber,.............. | 5. | Vulcanizes in three hours, 300° F. |

The above are all sold free of tariff to any person who wants them.

The American Hard-Rubber Company's Gum, $3 per pound. Vulcanizes in 3 hours at 300° F. This is sold only to those holding license from the company.

VULCANIZERS.—Cast Iron to be used on any common stove, with flasks and tools complete, $15.

Roberts' for gas or alcohol, for 2 pieces $18, 3 pieces, $20. *Flasks and tools extra.*

New improved Copper Machines, for 3 pieces with flasks and scrapers complete $25.

Extra Flasks, ordinary...... 35 cents. Improved............. .... 40 cents.

" Scrapers. " ...... 25 " " .................. 35 "

Thermometers.......................................................... $3 00

Full and complete printed instructions will accompany each machine.

Practical Instructions.................................................. 10 00

JNO. T. TOLAND,

Dental Depot, 38 West Fourth Street, Cincinnati, Ohio.

N. B. I think it probable that I have *less real interest* in this matter than any dentist in the U. S., being equally ready to supply a license and materisls to those who wish, or to supply machines and materials without a license to those who do not want a license. But whilst I am ready to acknowledge the *just* claims of all persons, I wish at the same time to protect the interests of the profession by affording them such information as will enable them to act understandingly.

Crystal or Sponge Gold.—I have just received a supply of this article from the patentee and manufacturer, which is most beautiful in appearance, and works to a *charm*. Those who desire to use this article, may hereafter depend on receiving it promptly, as I have made an arrangement which will ensure a constant supply.

---

The Artisan and Blowpipe.—I would call attention to this new apparatus, as advertised and illustrated in our advertising pages. It is one of the most perfect contrivances ever placed before the profession. Those who have tried, speak of it in the highest terms.

---

Photographs.—At the request of the present graduating class, I have procured a beautiful photograph of each of the members of the faculty of the Ohio College of Dental Surgery all upon one plate 10 by 12½ inches. It is finely gotten up, and makes a neat picture. The pictures are all faithful representations. They can be obtained by applying to, or addressing John A. Watling, care of J. T. Toland, 38 West 4th Street, at $2 50 per copy.

J. A. WATLING.

THIRTEENTH VOLUME

OF THE

### DENTAL REGISTER OF THE WEST,

EDITED BY

J. TAFT and GEO. WATT.

**Issued Monthly. - - - $3 a year in Advance.**

The Volume commences in July. CONTRIBUTIONS to the pages of the Register respectfully solicited.

Exchanges and Correspondents will please address "Dental Register," or J. TAFT, Cincinnati, O; Business communications to JNO. T. TOLAND.

The Register being issued Monthly is always fresh, and in advance of all others, therefore indispensable to the practitioner who desires to keep posted up with the new inventions and improvements which are daily developed.

Neither expense nor effort will be spared to give value and variety to its contents. Articles will be illustrated with well executed engravings, whenever they will add to the interest or assist in explanation.

Its extensive circulation and frequency of issue makes it the BEST DENTAL ADVERTISING MEDIUM in the United States.

### TERMS OF ADVERTISING.

| | One Year. | 6 Mo. | 3 Mo. | 1 Insertion. |
|---|---|---|---|---|
| One Page, | $50 | $30 | $20 | $15 |
| Half. | 30 | 20 | 15 | 10 |
| Quarter. | 20 | 15 | 10 | 7 |
| One-Eighth. | 15 | 10 | 7 | 5 |

**JNO. T. TOLAND, Pub. and Proprietor,**

38 *West Fourth Street, Cincinnati, Ohio.*

